# Issues in the Implementation of Directive 2013/35/EU Regarding the Protection of Workers against Electromagnetic Fields

**DOI:** 10.3390/ijerph182010673

**Published:** 2021-10-12

**Authors:** Gian Marco Contessa, Simona D’Agostino, Rosaria Falsaperla, Carlo Grandi, Alessandro Polichetti

**Affiliations:** 1 Fusion and Technology for Nuclear Safety and Security Department, National Agency for New Technologies, Energy and Sustainable Economic Development (ENEA), 00044 Frascati, Italy; 2 Department of Occupational and Environmental Medicine, Epidemiology and Hygiene, Italian Workers’ Compensation Authority (INAIL), 00078 Monte Porzio Catone, Italy; s.dagostino-sg@inail.it (S.D.); r.falsaperla@inail.it (R.F.); ca.grandi@inail.it (C.G.); 3 National Center for Radiation Protection and Computational Physics, Italian National Institute of Health (ISS), 00161 Rome, Italy; alessandro.polichetti@iss.it

**Keywords:** electric and magnetic fields, health effects, magnetic resonance imaging, occupational exposure

## Abstract

In 2016 the Directive 2013/35/EU regarding the protection of health and safety of workers exposed to electromagnetic fields was transposed in Italy. Since then, the authors of this paper have been faced with several issues related to the implementation of the provisions of the Directive, which pose some interpretative and operative concerns. A primary critical feature of the Directive is that, in some circumstances, conditions of “overexposure”, i.e., of exceeding the exposure limits, are allowed. In the case of transient effects, the “flexibility” concerning the compliance with exposure limits is based on the approach introduced by ICNIRP in its guidelines on static magnetic fields and on time-varying electric and magnetic fields. On the contrary, the possibility of exceeding the exposure limits for health effects, formally recognized in the article of the Directive dealing with derogations, is not included in the ICNIRP guidelines. This paper analyzes the main concerns in interpreting and managing some provisions of the Directive with particular reference to the issue of how the employer can manage the situations of overexposure.

## 1. Introduction

Directive 2013/35/EU, regarding the protection of health and safety of workers exposed to electromagnetic fields (EMFs) [1], presents an innovative safety approach that involves an important adaptation of the professionals involved in the field from an organizational and cultural point of view.

One of the main innovative aspects, which undoubtedly presents some difficulties of application, is that, in specific cases and in duly justified circumstances, conditions of “overexposure”, i.e., the exceeding of the exposure limit values (ELVs) set out in the Directive, are allowed.

Strictly connected to the possibility of overexposures is the distinction between health effects ELVs, above which workers might be subject to adverse health effects (such as thermal heating or stimulation of nerve and muscle tissue), and sensory effects ELVs, above which workers might be subject to transient disturbed sensory perceptions (such as vertigo, nausea and magnetophosphenes) and minor changes in brain functions; this approach is consistent with that introduced by the International Commission on Non-Ionizing Radiation Protection (ICNIRP) in its guidelines for static magnetic fields [2] and for time-varying electric and magnetic fields in the range 1 Hz–100 kHz [3].

Consistently with ICNIRP, the Directive has adopted a “flexible” approach regarding the compliance with the sensory effects ELVs (Article 3). According to this approach, transient effects on sensory perceptions and possible changes in brain functions are regarded as “minor” as they do not have any known long term or serious health consequences, but in some circumstances may pose safety risks. The eventuality that a worker can experience them is accepted if the consequences for safety are controlled. In any case, the condition of overexposure with respect to sensory effects ELVs assumes an exceptional nature and it must be: (1) justified by the work practice or process; (2) temporary during the work shift and (3) accompanied by the adoption of specific and additional protective measures by the employer.

Moreover, the Directive goes beyond the intentions of ICNIRP introducing in Article 10 (“Derogations”) the possibility of exceeding even the health effects ELVs. This option was introduced primarily to limit the possible impact of restrictions on magnetic resonance imaging (MRI), so as not to hold back the development of this medical practice of undoubted benefit for patients. This is in anticipation of the technological evolution of tomographs and the implementation of practices that could involve higher exposure levels for the operators. Nevertheless, the provisions relating to the derogations may be extended by the Member States to other specific sectors or activities.

However, in the spirit of the Directive, the possibility of derogations must not in any case exempt the employer from assessing the risk and taking all the necessary measures in order to avoid any adverse effect to the health and safety of the workers, so preserving an effective protection of workers. Therefore, a key point to understand is how the employer can demonstrate, as required by the Directive, that workers are still protected against adverse health effects even when the ELVs set to protect against these effects are exceeded. For this purpose, it would be necessary to answer the following questions: in relation to how much the health effects ELVs are exceeded, which effects could be expected? Are these effects cause of discomfort or of real health risks for the workers concerned?

Another critical issue is the risk assessment and management of workers at particular risk (workers with medical devices, implanted or worn, and pregnant workers) for which a shared approach is not yet available at the international level. In any case, workers at particular risk are excluded from the application of both the flexible approach and derogations as in their case the management of the risk is based on the application of the restrictions established for the protection of the population. For this reason, this topic will not be discussed in the following.

This paper analyzes the main concerns in interpreting and managing some provisions of the Directive. In particular, the issue will be addressed of how the employer can manage the situations of overexposure.

## 2. Issues Concerning Flexibility for Sensory Effects Exposure Limit Values (ELVs)

According to ICNIRP guidelines, as mentioned, Directive 2013/35/EU distinguishes between sensory effects and health effects ELVs.

Sensory effects ELVs are aimed to prevent effects such as vertigo, nausea, retinal phosphenes, metallic taste and possible minor changes in some brain functions induced by static magnetic fields (SMFs) and low frequency electric and magnetic fields (up to 400 Hz) [2,3], and auditory effects of pulsed radiofrequency fields between 300 MHz and 6 GHz [4]. These effects are transient and may pose safety risks depending on both the type of activity carried out by the worker and the environment where this activity is carried out.

On the contrary, health effects, such as peripheral nerve stimulation, involuntary muscle contraction and cardiac stimulation up to the induction of ventricular fibrillation, are regarded as potentially dangerous for health, although they include minor effects such as the simple perception of the electric currents induced at the body surface by external fields.

The possibility of exceeding the limits represents a novelty in terms of protection from exposure to electromagnetic fields. In particular, the flexibility of sensory effects ELVs refers to conditions in which the potential temporary exceeding is not accidental but is foreseen by the employer who deems it necessary for the purposes of the practice or the production process. In any case, compliance with specific conditions stated by the Directive must be assured. Moreover, it should be noted that, since these effects are in any case a source of discomfort for the worker, the Directive requires that the exceeding of the ELVs for sensory effects is only temporary.

Since sensory effects of static and low-frequency fields up to 400 Hz occur at lower exposure levels with respect to health effects, the pertinent, flexible, limits are more restrictive than the rigid limits established for protection from health effects, differently from the case of pulsed radiofrequency fields, as explained later.

In the case of exposure to SMFs, the Directive establishes a sensory effects ELV of 2 T “related to vertigo and other physiological effects related to disturbance of the human balance organ resulting mainly from moving in a static magnetic field”. Actually, sensory effects such as vertigo and nausea are probably due to motion-induced electric fields, i.e., induced according to Faraday’s law by the temporal variations of the SMF perceived by a moving subject [5]. However, the Lorentz force on ionic currents in the vestibular organ could contribute to them also in a stationary subject exposed to a SMF.

The Directive indicates the control of movements as a specific preventive measure in the event of exceeding the sensory effects ELV. This is particularly relevant in the case of the MRI where, currently, one of the most relevant issues concerns the movement in the SMF, an aspect that involves any operator accessing the scanning room [6,7,8].

According to the Directive, the European Commission should have adopted a delegated act to insert into Annex II of the Directive itself, fixing the ELVs for the non-thermal effects in the range 0–10 MHz, the ICNIRP guidelines for limiting exposure to electric fields induced by movement of the human body in a SMF and by time-varying magnetic fields below 1 Hz, as soon as they were available. However, even if these guidelines were published in 2014 [9], the aforementioned delegated act has never been adopted by the EU Commission.

Therefore, the Directive does not require to assess specific physical parameters designed to limit the internal electric fields induced by the movement of the subject within a SMF, but only to verify the value of the external magnetic induction.

However, the control of movements, i.e., the capacity of the operator to move slowly, avoiding sudden movements and rotations (even of the head), has to be considered among the protective and preventive measures useful to reduce or eliminate sensory effects and transient symptoms in cases where the 2 T ELV is exceeded, if the conditions set out in the flexibility system are met.

Doubts have often been expressed to the authors of this paper on the mandatory application of the 2014 ICNIRP guidelines and, therefore, on the obligation to carry out complex evaluations, including, where appropriate, dosimetric evaluations. Since the recommendations indicated in these guidelines are not within the requirements of the Directive, they can just be applied on a voluntary basis to improve the risk assessment.

Regarding low-frequency fields, the potentially most relevant sensory effect is represented by the retinal phosphenes. Unlike the SMF, for low-frequency fields behavioral measures (e.g., movement control) are not applicable and worker’s awareness, deriving from an appropriate information and training activity, is the key point to prevent safety risks.

Concerning the auditory effects of pulsed radiofrequency fields, the pertinent limits are flexible as well, but not necessarily more restrictive than the limits for health effects of radiofrequency fields; actually, limits for auditory effects are expressed in terms of specific absorption (SA, J/kg), while limits for health effects of radiofrequency fields are expressed in terms of specific absorption rate (SAR, W/kg), and these two physical quantities cannot be compared.

However, the possibility of auditory effects is limited to very particular exposure scenarios, for example the case of exposure to pulsed radar emissions, and can be prevented by adopting technical and organizational measures. The marginality of these effects is confirmed by the fact that the recent ICNIRP Guidelines, published in 2020 [10], do not take them into consideration.

In any case, for all the frequency ranges, and for the generality of sensory and other minor transient effects, information and training are of paramount importance, as repeatedly outlined in the Directive, especially in Article 6.

Anyway, it is useful to report some additional considerations on transient effects given the importance of the flexible approach in the Directive. Although these effects are not fully established in terms of number and types, some of them (sensory effects such as vertigo/dizziness, retinal phosphenes, nausea and metallic taste) show consolidated evidence regarding the fields involved and the induction thresholds. On this basis, the regulation in force establishes exposure limits, although the scientific evidence would suggest that, at least for vertigo and dizziness in association with SMF exposure, the induction threshold could sometimes be lower than the widely recognized 2 T [11].

In general, transient effects are expected to occur in workers operating in the controlled area of diagnostic MRI sites or in occupational settings involving exposure to EMF of frequency <400 Hz emitted by sources like welding machines, high power electric engines or circuitry carrying large electric currents.

In these occupational settings, some other transient effects, not yet completely established by research activity, cannot be excluded, e.g., small cognitive alterations, as the central nervous system has generally stimulation thresholds lower than the peripheral one. Moreover, the central nervous system has the capacity to integrate and amplify electric stimuli, making the question of thresholds more uncertain [12]. The occurrence of transient effects is limited to a fraction of the exposed subjects depending on individual features. However, the number of affected subjects tends to grow as the exposure increases. Higher exposures could also result into a higher intensity of symptoms in subjects already showing the effects [13]. These effects are reversible: they cease as exposure ceases. This was observed in most subjects belonging to a group of physicians working in the “controlled” zone of diagnostic MRI sites (1,5 and 3 T) showing one or more transient effects [14,15]. The repeated experience of the effects due to repeated exposures during the work shift may often result in a decrease of symptoms in some subjects, until their disappearance in relatively short time periods (few weeks), suggesting an adaptation over time [15].

In given exposure situations, transient effects may compromise the safety of the exposed subjects or, in some cases, of other subjects (see for instance [2]). One of the most indicative examples is given by the occurrence of vertigo or dizziness during the execution of critical tasks (such as patient care during a MRI examination). However, small alterations of sensory perception may also affect attention and reaction times, having the potential to interfere with the execution of particular tasks. As a consequence, workers have to be adequately informed on the effects, on the possible consequences in terms of annoyance and safety, and on measures and behavior to reduce their occurrence. Moreover, health surveillance is provided.

Current evidence does not support health risks arising from transient effects occurrence, but a significantly impact on the psychophysical wellbeing of a repeated experience of one or more of these effects during work shifts for a prolonged period may not be excluded. The adaptation to transient effects, involving an attenuation and even the disappearance of the related symptoms after repeated exposure, is reported in the literature but is not likely to involve all exposed workers [15]. For a subset of them, personality features or psychiatric conditions (such as anxiety or psychotic traits) might result in an increased impact of transient effects due to EMF exposure, especially if these effects occur repeatedly. The perception of a transient effect may in fact be amplified and could contribute to trigger and/or worsen the clinical features linked to the medical condition of the subject. At the moment, the aforementioned considerations are on a theoretical level, but it would be important to explore this topic more deeply. In this regard, the planning of multicentric cohort studies with a careful characterization of EMF sources and exposure levels, accompanied by a detailed individual health assessment, is likely to represent the most suitable approach.

## 3. Issues Concerning Derogations

As the flexible approach with regard to sensory effects ELVs was not considered sufficient in order to limit the impact on certain activities, Article 10 of the Directive introduces the possibility of derogations from compliance with the ELVs. Notwithstanding that Article 10 of the Directive does not specify which type of ELVs are its subject, it appears that derogations regard primarily the health effects ELVs as the exceeding, albeit temporary, of sensory effects ELVs is allowed under the flexible approach outlined by Article 3. However, even sensory effects ELVs could fall within the scope of Article 10 if their exceeding is not temporary during the work shift. As a consequence, for sensory effects ELVs the employer’s choice between the implementation of the flexible approach or the recourse to derogations depends on the temporal pattern of overexposure within a work shift. However, neither the Directive nor (for instance) its Italian transposition give additional indications on what is meant by temporary, raising at least two questions: how many times may ELVs be exceeded in the work shift? What is the maximum duration allowed for the single exceeding? This is a critical point when implementing provisions of the Directive and, to our knowledge, it has not yet been fully addressed by guidelines or good practices.

Moreover, it has to be outlined that overexposures with respect to health effects ELVs imply simultaneously exceeding the lower sensory effects ELVs (leaving aside auditory effects, for the reasons described above) and, as a consequence, there is a real risk that a health effect adds to one or more transient effects whose frequency and intensity could increase as the level of exposure increases (as previously discussed for the SMF). Therefore, in the case of derogations from compliance both to sensory and health effects ELVs, a strict control of transient effects is mandatory.

Although the possible impact on the medical practice of MRI was the main reason behind the introduction of derogations, the derogation system can also be applied by Member States to other specific activities. Different working sectors can be interested in this provision, both in the industrial (e.g., the welding sector) and in the medical field (for example transcranial magnetic stimulators and electrosurgical units), where the possibility of exposures exceeding the limits for health effects and the difficulty of keeping these exposures below the ELVs (without incurring in excessive costs) are known [16,17,18,19].

The possibility of derogations from compliance to ELVs is strongly conditioned as it requires the implementation, by the employer, of a series of fulfillments documenting that an effective and efficient risk assessment has been carried out and that workers are still protected against adverse health effects and safety risks. Then, the critical issue arises of what measures the employer can implement to ensure the health and safety of workers in the event of exceeding the ELVs for health effects.

Regarding the installation, testing, use, development, maintenance of or research related to MRI equipment for patients in the health sector, the Directive indicates to ensure that the instructions for safe use provided by the manufacturer in accordance with Council Directive 93/42/EEC of 14 June 1993 [20] concerning medical devices are followed. It has to be noted that the manufacturer has the possibility to declare that the product (the MRI system) is in accordance with the essential requirements of Council Directive 93/42/EEC, including the health and safety requirements for the users, if (but not only if) the prescriptions of the relevant harmonized technical standards have been followed. In the case of MRI, the European standard EN 60601-2-33, “Medical electrical equipment—Part 2-33: Particular requirements for the basic safety and essential performance of magnetic resonance equipment for medical diagnosis”, which includes also the prescriptions relative to the instruction for use, is particularly noteworthy [21,22]. Therefore, it is a key point to understand how the requirements of the EN 60601-2-33 can assure the protection of workers against electromagnetic fields.

As regards derogations for specific sectors or activities different from MRI for patients in the health sector, and from military applications for which Member States may allow other specific protection systems, the employer can use more specific and internationally recognized standards and guidelines to demonstrate that workers are still protected.

Ensuring the protection of workers against health and safety risks arising from exposure to electric, magnetic and electromagnetic fields is the key concern when a derogation from compliance with ELVs is authorized and applied. The question is challenging, involving the type of exposure, the biological and/or dosimetric rationale supporting the ELVs as well as individual and environmental circumstances.

The approach to manage health and safety requirements when a derogation is in place may be different for static fields, low-frequency fields and high-frequency fields as will be discussed in the following paragraphs and summarized in Table 1.

### 3.1. Static Magnetic Fields

The health effect ELV for SMFs, relative to controlled working conditions, laid down by the Directive 2013/35/EU, is equal to 8 T, as recommended by ICNIRP in 2009 [2]. Actually, based on an analysis of known action mechanisms, ICNIRP states that the major potential concerns with respect to limiting exposure to SMFs are cardiovascular and neurological effects, but studies on humans exposed to SMFs up to 8 T do not provide evidence of any irreversible or serious adverse health effects. ICNIRP recommends restricting exposure below 8 T because for higher exposures there is no human experience and therefore there is lack of knowledge.

As mentioned above, according to the Article 10 of the Directive 2013/35/EU, exposures may exceed the health effects ELV if the employer demonstrates that workers are still protected against adverse health effects. However, at the present time, on the basis of the current lack of knowledge about the effects of SMFs above 8 T, it is not possible to guarantee the health and safety of workers exposed to these levels as requested by the derogations system.

In the case of MRI, it is necessary to ascertain if the technical standard EN 60601-2-33 [21,22] contains useful information about protection of workers against the adverse health effects of SMF levels higher than 8 T. According to EN 60601-2-33, the instructions for use should explain the health effects related to the increased SMF and that adequate training shall be given to MRI workers to minimize them.

However, consistently with current scientific knowledge, no information is available about minimizing the health effects of SMFs above 8 T, and the technical standard EN 60601-2-33 does not provide other indications about how to manage an exceeding of the health effects ELV (which is possible if in the examination room the SMF is higher than 8 T in points accessible to workers). For this reason, merely following the instructions for safe use drawn up by the manufacturer according to EN 60601-2-33 does not guarantee that workers are still protected against adverse health effects and derogations from the compliance to health effects ELV for the SMFs cannot apply just on this basis.

Anyway, the authors are not aware of any occupational settings (included the controlled zone of high fields MRI facilities in health care units) involving the partial or total body exposure of the workers to SMFs exceeding 8 T. Similarly, even non-temporarily exceeding the 2 T sensory effects ELV for SMFs exposure might not easily occur for workers, even those operating in MRI facilities.

### 3.2. Low-Frequency Electric and Magnetic Fields

Derogations regarding the non-thermal effects of low frequency electric and magnetic fields (1 Hz–10 MHz) require to take into account the following considerations.

Basic restrictions, corresponding to the health effects ELVs, have been derived by ICNIRP from biological thresholds of stimulation of excitable tissues introducing reduction factors to compensate for various sources of uncertainty, such as the extrapolation of animal data to effects on humans, differences in physiology and tolerance of different people, and statistical uncertainties in the dose-response function.

In the low-frequency range, experimental data indicate that the stimulation threshold of peripheral nerve fibers falls in the 4–6 V/m range of electric field strength [3]. Stimulation thresholds of both skeletal and myocardial muscle cells, although variable, are significantly higher than those of nerve fibers.

When deriving the basic restriction for workers, a reduction factor of 5 has been applied to the minimum identified stimulation threshold in order to account for the uncertainties described above. Obviously, a mere exceeding of the basic restriction/ELV does not imply necessarily a nerve stimulation; however, when considering a specific individual exposed to the field, we cannot exclude that, for her/his particular sensitivity, a stimulation effect occurs at exposure levels between the ELV and the identified threshold. Therefore, it is not possible to guarantee the health of workers exposed at levels exceeding the health effects ELV for low-frequency fields, as requested by the derogations system, just on the basis of the use of reduction factors by ICNIRP.

For this purpose, it is necessary to consider that the lowest biological effect threshold corresponds to the mere perception of the electric currents induced by external low-frequency fields at the body surface. It has also to be considered that the thresholds of painful perceptions, and of potentially dangerous effects, such as involuntary muscle contraction and cardiac stimulation up to the induction of ventricular fibrillation, are increasingly higher. Protection of workers could be assured if it is guaranteed that just minor effects like non-painful perception can occur.

Some useful indications to this end are provided in the European standard EN 60601-2-33 [21] with regard to the specific sector of MRI.

For both low frequency and radiofrequency fields (discussed in the following section), the European standard EN 60601-2-33 extends to MRI workers the same exposure limits established for patients, implying that workers could experience some minor biological effects, provided that this eventuality is balanced by adequate training. The rationale of this choice can be found in the essential requirements of Directive 93/42/EEC (Annex I), where it is stated that the devices must be manufactured in such a way that any risks (not only for patients, but also for users) which may be associated with their intended use are acceptable when weighed against the benefits to the patient (Directive 93/42/EEC is repealed by the Regulation (EU) 2017/745 of the European Parliament and of the Council of 5 April 2017, but the essential requirements established by the Directive are not changed) [20,23].

On the contrary, the basic restriction of ICNIRP were set without considering the need to balance health risks of MRI workers with benefits to the patient, because social (and economic) considerations are outside of the remit of ICNIRP.

Although this approach can be considered debatable, it nevertheless represents a reference for the employers. On the other hand, the instructions for safe use provided by the manufacturer should include information on highest levels of exposure in areas accessible to MRI workers along with a description of ways to mitigate the risks related to the exposure.

The standard EN 60601-2-33 defines three different operating modes (normal, first level controlled and second level controlled) at which different prescriptions for patient’s safety have to be applied. At these different operating modes defined for patients, different prescriptions are foreseen even for worker’s protection.

For low-frequency magnetic fields, associated to the switching of gradient coils in the MRI equipment, the standard sets one unique exposure limit for all the operating modes to prevent cardiac stimulation, while limits set to minimize peripheral nerve stimulation (PNS) are differentiated for normal and first-level controlled operating modes, but no limits are set for the second level controlled operating mode.

The limit used in the standard to avoid cardiac stimulation incorporates a safety factor of 3 below the 1-percentile threshold reported in literature [24,25]. According to the standard, this limit would correspond to a likelihood for cardiac stimulation lower than 2 × 10^−9^. In addition, it turns out that the peripheral nerve stimulation limit given in the standard is below the cardiac stimulation limit for all stimulus durations of practical interest.

Limits related to PNS are defined with reference to a PNS threshold corresponding to the onset of the activation of the nervous system due to gradient switching. This threshold can be directly determined by the manufacturer by experimental work with volunteers in a representative model of the gradient unit or, as an alternative, on the basis of a numerically defined limit provided in the standard itself and derived from the median threshold for perception observed in human studies reported in literature. Nyenhuis et al. [26] found that the exposure level for the lowest percentile for uncomfortable stimulation is approximately equal to the median perception threshold (corresponding to the PNS threshold in the European standard). Moreover, the lowest percentile for intolerable stimulation occurs at an exposure level approximately 20% above the median perception threshold. For these reasons, the European standard states that, for normal operating mode, the gradient system shall operate at a level that does not exceed 80% of the PNS threshold level, and for the first level controlled operating mode the gradient system shall operate at a level that does not exceed 100% of the PNS threshold level.

As regards MRI workers protection, the standard states that the exposure limits are the same as the maximally allowed limits for the patients. Considering that for the second-level controlled operating mode a limit for the minimization of occurrence of intolerable PNS in the patient is not defined, the authors believe that even in this operating mode the worker cannot be exposed at levels higher than the limit for the first level controlled operating mode. If we apply to workers this limit, corresponding to the median perception threshold, we accept that up to 50% of exposed subjects can sense the passage of electric currents induced by the magnetic field and up to 1% can feel uncomfortable sensations. By contrast, we can confidently exclude the possibility of intolerable stimulation, which lowest percentile occurs at exposure levels approximately 20% above the limit.

Derogations from compliance to health effects ELVs for low frequency fields could apply in the case of MRI equipment for patients in the health sector if the instructions for safe use provided by the manufacturer, which have to be followed, are in accordance not only with Council Directive 93/42/EEC, but in particular with the European standard EN 60601-2-33 [21]. Actually, by following the European standard, severe effects like cardiac stimulation are prevented, the possibility of intolerable stimulations is confidently ruled out, and the only foreseen effects are of minor importance consisting just in perceptions.

As regards other sectors or specific activities different from MRI, the above considerations should be addressed in specific technical standards, taking into account that an analogous balance of health risks for workers with benefits to the patient could be considered in the case of other medical applications.

### 3.3. Radiofrequency Fields

As in the case of low-frequency fields, basic restrictions for thermal effects of radiofrequency fields (100 kHz–300 GHz) have been derived by ICNIRP from biological thresholds introducing reduction factors to compensate for uncertainties. Only thermal effects will be taken into account in the following, leaving out effects like dielectric rupture of biological membrane, unrealistic in occupational exposure situations.

As regards the possibility of derogations from compliance to health effects ELVs, the first exposure situations that we consider are whole-body exposures. The relevant health effect ELV in these situations, related to whole-body heat stress, is the SAR averaged in the body over any six-minute period, equal to 0.4 W/kg as established in the 1998 ICNIRP guidelines for occupational exposures [4].

This value was obtained applying a reduction factor of 10 to a threshold of 4 W/kg corresponding to a body temperature increase of about 1 °C after about 30 min of exposure in a moderate thermal environment, as observed in resting volunteers exposed to EMFs generated by MRI scanners [27,28]. Actually, established biological and health effects in the frequency range from 10 MHz to a few GHz are consistent with responses to a body temperature rise of more than 1 °C: in vitro and in vivo studies have reported a number of biological effects involving neurobehavioral, immune, endocrine and reproductive functions as well as changes in cell morphology, water and electrolyte content, and membrane functions at EMF exposure levels capable to induce temperature rises in excess of about 1–2 °C [29].

Similarly to the low-frequency case, merely exceeding the ELV of 0.4 W/kg, which under normothermic conditions guarantees that body core temperatures do not rise more than about 0.1 °C, does not imply necessarily health effects due to thermal stress; therefore, it is necessary to understand how much it would be possible to exceed this ELV with no health risks for the exposed workers. The new 2020 ICNIRP guidelines for limiting exposure to electromagnetic fields in the radiofrequency range, not yet implemented in the European regulation, may be a useful, updated, scientific reference in this respect [6]. These guidelines are a revision of the 1998 guidelines [4] as regards radiofrequency fields, taking into account the advances in radiofrequency dosimetry, the thermophysiological data accumulating in the last 20 years and the necessity to adapt the protective requirements to new radiofrequency sources, like 5G telecommunication systems. These guidelines introduce new physical quantities and new restrictions to address the protection from superficial absorption of radiofrequency in the range 6–300 GHz and from rapid temperature rise for local exposures. However, the restrictions concerning the whole-body SAR and the local SAR are not changed with respect to the 1998 guidelines (and, as a consequence, with respect to the Directive 2013/35/EU), being respectively equal to 0.4 W/kg (whole body SAR), 10 W/kg (localized SAR for head and torso) and 20 W/kg (localized SAR for limbs). ICNIRP 2020 guidelines not only confirm but also enforce the rationale underlying these restrictions.

As reported in the rationale of these new guidelines, in the range 100 kHz–6 GHz an increase of 1 °C in body core temperature requires exposures of at least 1 hour involving a whole-body average SAR of 6–8 W/kg in thermoneutral conditions (28 °C of ambient temperature for the resting naked body) in healthy individuals [30]. A temperature rise of 1 °C may take longer (even a further 100 min) in the case of heavy sweating. By contrast, elderly people, who have a reduced sweating rate, needs a SAR of just 4.5 W/kg to increase their body core temperature of 1 °C after 1 h exposure [30]. Therefore, the restriction of whole-body SAR of 0.4 W/kg is highly protective, not only including a reduction factor of 10 but applying the latter to the most conservative estimate of the whole-body SAR requested for a 1 °C increase in body core temperature. Moreover, while this SAR value was averaged over 6 min in the previous guidelines, in 2020 guidelines it is averaged over a 30 min time period, which is a conservative estimate with respect to 1 hour of exposure suggested by dosimetric models to reach the steady state increase of 1 °C in body temperature, so both 6 min and 30 min averaging times represent an additional conservative parameter.

Considering that the body core temperature is not constant during the day, and can vary by some tenths of a degree, it can be reasonably assumed that, in case of derogations, an increase of about 0.5 °C does not pose real risks for workers. Results of dosimetric studies suggest that this limitation in temperature rise can be assured by limiting the whole-body average SAR to about 2 W/kg, excluding the case of subjects with a reduced sweating rate [30].

Support for this view is given by provisions of the European standard EN 60601-2-33 for MRI [21]. For protection of the patient, in fact, the standard provides a whole-body SAR limit of 2 W/kg for the normal operating mode, of 4 W/kg for the first level controlled operating mode and no limits for the second-level controlled operating mode. Moreover, for the first-level controlled operating mode the standard indicates to reduce the whole-body SAR limit as the environmental temperature increases. Specifically, a reduction of 0.25 W/kg per degree is indicated, starting from 4 W/kg at 25 °C (thermoneutral condition of human body wearing clothes) until a value of 2 W/kg is reached at 32 °C. Exposure limits for MRI workers are the same as the maximally allowed limits for patients; as in the case of low-frequency magnetic fields, it can be reasonably assumed that in the second-level controlled operating mode, where no limits are indicated, MRI workers cannot be exposed at levels higher than the 4 W/kg limit for the first-level controlled mode. However, on the basis of previous considerations, the authors believe that the MRI worker, in case of derogations, should not be exposed to levels higher than the 2 W/kg limit for the normal operating mode.

Moving back to ICNIRP 2020 guidelines [10], it is noteworthy that they provide some considerations to mitigate risk for occupational exposure, pointing out, for instance, other factors which may affect body core temperature such as heat sources in the workplace and the use of thermally insulating clothing by workers. They also mention concerns for high physical activity and certain medical conditions.

In summary, current scientific evidence does not indicate any real risk of adverse health effects for radiofrequency exposure resulting in a whole-body SAR up to 2 W/kg, implying a body core temperature rise of about 0.5 °C, in healthy subjects in thermoneutral conditions. Moreover, diseases critically altering thermoregulation of the body are often too severe to be compatible with the ability to perform a number of jobs, including jobs implying the exposure to radiofrequency in medical, industrial or telecommunication sectors. Drugs acting on cardiovascular or nervous system (e.g., antihypertensive drugs, antiarrhythmic drugs, antipsychotics etc.) are very unlikely to severely affect thermoregulation in moderate thermal environments or during moderate physical activity. On the other hand, the elderly, whose thermoregulatory mechanisms are less efficient, are no longer part of the working population. As a consequence, in case of derogations to compliance to the ELVs a relaxation of whole-body SAR up to 2 W/kg could be considered acceptable, since this is a value implying only a slight thermal stress even if maintained for prolonged periods, unless the worker operates in severe thermal environments or wears thermal insulating clothes. If this is the case, a more detailed risk assessment is required.

With regard to the ELVs for localized SAR, i.e., 10 W/kg for head and trunk and 20 W/kg for limbs, averaged on a time period of 6 min and up to 6 GHz, derogation to compliance is more difficult to be scientifically supported. The most updated scientific reference is, once again, represented by the 2020 ICNIRP guidelines [10]. In fact, basic restrictions to the localized SAR are the same as in the Directive 2013/35/EU. The operational threshold for adverse effects is a steady state temperature increase of 2 °C for head and trunk and 5 °C for limbs. A restriction of 10 W/kg ensures that the temperature rise of head and trunk tissues is within 1 °C, while a restriction of 20 W/kg involves a temperature increase in the limbs less than 2.5 °C, therefore these two local SAR restrictions incorporate a reduction factor of 2. These restrictions are conservative since the operational thresholds of 2 °C and 5 °C of temperature rise do not allow the tissue temperature of 41 °C to be achieved, which is the tissue damage threshold, when the basal temperature is within the physiological range (37–38 °C for head and trunk and <36 °C for limbs). The operational threshold of 2 °C is applied by ICNIRP also to testes, the basal temperature of which is substantially lower than that of other tissues of head and trunk, keeping their temperature below about 37 °C. It should be noted that a temperature increase up to 2 °C (which may also be due to normal activities such as sitting, or other conditions like wearing tight trousers) may result in reversible changes to sperm function. However, according to ICNIRP, there is currently no evidence that such effects are sufficient to impair health [10].

Moreover, the time average of 6 min is conservative as dosimetric data indicate that up to 90% of the steady state temperature is reached after 15 min of exposure. Additionally, the 2020 ICNIRP guidelines state that occupational exposure of the limbs is unlikely to increase local temperature by more than 2.5 °C.

However, the reduction factor 2 for derivation of local SAR restrictions leaves a narrow margin of safety. Moreover, the basal temperature of limbs is more dependent on the ambient temperature than the core temperature of head and trunk, representing a concern for workers exposed to radiofrequency in severe thermal environments.

Therefore, current scientific data on localized SAR give a weaker support to derogations from compliance with ELVs with respect to those related to whole body SAR. It has to be noted that the European standard EN 60601-2-33 prescribes for the patient, and therefore also for MRI workers, the same local SAR limits of the Directive, as long as the normal operating mode is concerned. For the first level controlled operating mode, the local SAR values are doubled, completely neglecting the reduction factor and therefore making it difficult to ensure the protection of exposed workers. For this reason, the authors believe that, as regards localized exposures, the European standard does not provide useful indications for possible derogations from compliance to local SAR ELVs.

**Table 1:** Main findings about the possibility of derogations for the different frequency ranges.

	Overexposure Conditions	Literature Indications	Possibility of Derogations
Static magnetic fields	>8 T	For exposures > 8 T there is no human experience and therefore there is lack of knowledge [2].	It is not possible to guarantee health and safety of workers.
Low-frequency electric and magnetic fields	>0.8 V/m * (1 Hz–3 kHz) >2.7 × 10^−4^ f V/m * (3 kHz–10 MHz)	The stimulation threshold of peripheral nerve fibers falls in the 4–6 V/m * range of electric field strength [3]. Human studies reported increasing thresholds for effects ranging from the mere perception of induced electric currents to uncomfortable and intolerable painful stimulations [26]. Thresholds for potentially dangerous effects (up to the induction of ventricular fibrillation) are increasingly higher [3]. In the case of Magnetic Resonance Imaging (MRI) equipment, the EN 60601-2-33 [21] extends to workers the limits set for patients. The limit set to avoid cardiac stimulation incorporates a safety factor 3 below the 1-percentile threshold reported in literature [24,25]. Compliance to the limits set to minimize peripheral nerve stimulation implies the possibility that up to 50% of exposed subjects can sense the passage of induced electric currents and up to 1% can feel uncomfortable sensations, while confidently excluding the possibility of intolerable stimulation [26].	It is not possible to exclude stimulation effects at exposure levels below the identified threshold in specific individuals for their particular sensitivities. Protection of workers could be assured if it is guaranteed that just minor effects like not painful perception can occur. Derogations from the compliance to health effects ELVs could apply in the case of MRI equipment if the instructions for safe use provided by the manufacturer, which have to be followed, are in accordance with EN 60601-2-33 the provisions of which allow prevention of severe health effects like cardiac stimulation while allowing just the possibility of minor effects like perceptions. Similar provisions should be provided in specific technical standards for other sectors or specific activities different from MRI, in particular in the case of medical applications where a balance of health risks for workers with benefits to the patient could be considered as in the EN 60601-2-33.
Radiofrequency fields	>0.4 W/kg (Whole-body)	Results of dosimetric studies suggest that a temperature rise < 0.5 °C can be assured by limiting the whole-body average SAR to about 2 W/kg, excluding the case of subjects with a reduced sweating rate [30].	Considering that the body core temperature is not constant during the day, and can vary by some tenths of °C, it can be reasonably assumed that, in case of derogations, an increase of about 0.5 °C does not pose real risks for workers and a relaxation of whole-body SAR up to 2 W/kg could be considered acceptable.
	>10 W/kg (Local SAR for head and trunk)>20 W/kg (Local SAR for limbs)	The local SAR limits are obtained applying a reduction factor of 2 to the effect thresholds to account for scientific uncertainty [10].	The reduction factor 2 for derivation of local SAR restrictions leaves a narrow margin of safety; therefore, it is not possible to guarantee health and safety of workers.

* r.m.s. values (in the European Directive 2013/35/EU the low frequency ELVs are reported in terms of peak values).

## 4. Conclusions

Among the issues introduced by Directive 2013/35/EU, the more critical applicative difficulties which an employer can face during implementation are related to the conditions of overexposure, and in particular to the necessity to ensure the health and safety of exposed workers.

A major issue discussed in this paper refers to transient effects in relation to the duration of exposures higher than the pertinent ELVs. This is a two-fold problem, since it concerns, on one hand, the interpretation of the concept of temporariness when the Directive states that ELVs are only temporarily exceeded, and on the other, the possible negative consequences on the worker’s health in the case of repeated, or long term, overexposures.

As regards the first problem, the authors are not aware of clear indications on what is meant by temporary neither in the Directive nor in subsequent documents, such as national transpositions of the Directive itself, guidelines or good practices. This is a critical point in implementing the provisions of the Directive because the employer can be face a need to choose between application of the flexible approach or a request to the competent authority of a derogation from compliance to the sensory effects ELVs. A clarification on this point is, therefore, needed.

With regard to the possible health implications of exposures exceeding the sensory effects ELVs, literature on the subject is scarce. Some indications derive from some observational studies on MRI workers, suggesting an adaption over time to this type of effects. However, the possibility remains of effects on psychophysical wellbeing due to a repeated experience of one or more of these effects during work shifts for a prolonged time period. In order to further investigate this topic, multicentric cohort studies with detailed individual exposure and health assessments are warranted.

The other critical issue discussed in this paper is how the employer can ensure that workers are protected even if the health effects ELVs are exceeded by derogations. This problem has been addressed separately for SMFs, low-frequency electric and magnetic fields and radiofrequency fields. The provisions of the standard EN 60601-2-33 relevant for MRI occupational exposures have also been discussed as regards their validity in protecting not only MRI workers but also workers in different sectors.

On the basis of the current lack of knowledge about the effects of SMFs exceeding the health effects ELV of 8 T, it is not possible to guarantee the health and safety of workers exposed to these levels as requested by the derogations system. Moreover, derogations from the compliance to the 8 T ELV would not be justified just on the basis of the observance, indicated in the Directive, of instructions for safe use of the MRI equipment.

On the contrary, as regards low-frequency fields, derogations from the compliance to health effects ELVs could apply in the case of MRI equipment if the instructions for safe use provided by the manufacturer, which have to be followed, are in accordance with the European standard EN 60601-2-33. The provisions of this standard make it possible to prevent severe health effects while allowing just the possibility of minor effects like perceptions. Similar provisions should be provided in specific technical standards for other sectors or specific activities different from MRI, in particular in the case of medical applications where a balance of health risks for workers with benefits to the patient could be considered.

Finally, as long as radiofrequency fields are concerned, it is necessary to distinguish between whole-body and localized exposures. In the first case, in the light of both the recent pertinent ICNIRP guidelines and the European standard on MRI, derogation from compliance to the ELV of 0.4 W/kg on the whole-body SAR could be considered acceptable up to 2 W/kg, a value implying only a slight thermal stress, unless the worker operates in severe thermal environments and/or wears thermal insulating clothes, requiring a more detailed risk assessment. For localized exposures, derogations from compliance to the local SAR ELVs, derived from the biological effects thresholds with a very small reduction factor, seem not to be justified.

## Data Availability

Not applicable.
